# Impact of Socioeconomic Characteristics on Metabolic Control in Children with Type 1 Diabetes in a Developing Country

**DOI:** 10.4274/jcrpe.galenos.2019.2019.0014

**Published:** 2019-11-22

**Authors:** Abeer Alassaf, Rasha Odeh, Lubna Gharaibeh, Sarah Ibrahim, Kamel Ajlouni

**Affiliations:** 1 University of Jordan Faculty of Medicine, Department of Pediatrics, Amman, Jordan; 2 University of Jordan Faculty of Medicine, Department of Clinical Pharmacy, Amman, Jordan; 3 University of Jordan, The National Center (Institute) for Diabetes, Endocrinology and Genetics, Amman, Jordan

**Keywords:** Type 1 diabetes, HbA1c, metabolic control, socioeconomic status, Jordan

## Abstract

**Objective:**

Adequate glycemic control in children with type 1 diabetes reduces the risk of future complications. Identifying factors affecting haemoglobin A1c (HbA1c) is crucial to management of metabolic control. We aimed to identify possible socioeconomic predictors of poor metabolic control this patient group in Jordan, a developing country with limited resources.

**Methods:**

Medical charts of children with type 1 diabetes attending the pediatric endocrine clinics in two major diabetes centers were reviewed. HbA1c ≥7.5% (58 mmol/mol) was considered to reflect poor metabolic control. Logistic regression analysis was performed to identify predictors of poor glycemic control. The association between socioeconomic characteristics and metabolic control was evaluated using multiple correspondence analysis (MCA).

**Results:**

Two hundred and fifty-nine children were enrolled in the study. One fifth of the patients (20.5%) achieved HbA1c <7.5%. Patients with dietary non-compliance [odds ratio (OR): 3.533, confidence interval (CI): 1.803 - 6.926; p<0.001], and those who were overweight (OR: 3.869, CI: 1.218 - 12.294; p=0.022) were more likely to have poor metabolic control. Children whose mothers had a bachelor’s degree or higher were less likely to have poor metabolic control compared to children whose mothers had only elementary education (OR: 0.241, CI: 0.079 - 0.734; p=0.012). MCA revealed an association between low socioeconomic status and poor metabolic control. Children with deceased mothers had significantly higher HbA1c of 10.6±1.86% compared to an average of 8.7±1.45% for the rest of participants (p=0.005).

**Conclusion:**

Low socioeconomic status, lower levels of maternal education and maternal death were associated with poor metabolic control. Identifying children with these risk factors might play an important role in optimizing metabolic control and provide better diabetes care.

**What is already known on this topic?**

Several predictors of type 1 diabetes mellitus outcomes in children have been studied, which include socioeconomic factors. A number of these social factors have been shown to negatively impact metabolic control including low socioeconomic status, single-parent family and inadequate parental supervision. There is a dearth of evidence on the impact of this type of factor in developing countries.

**What this study adds?**

Our study aimed to identify socioeconomic factors affecting metabolic control in children and adolescents with type 1 diabetes in a developing country. Identifying patients with high risk of poor metabolic control including those with low socioeconomic status and lower parental education level, will help in implementing early effective strategies for diabetes care and will result in a better metabolic control.

## Introduction

Type 1 diabetes is associated with microvascular and macrovascular complications, including diabetic retinopathy and nephropathy (1,2). The Diabetes Control and Complications Trial and the Epidemiology of Diabetes Interventions and Complications study showed that the progression of microvascular complications can be reduced by strict glycemic control (3). Adequate glycemic control, especially in the first five years of diabetes, slows the development of microvascular complications (4). These findings support the importance of maintaining a low haemoglobin A1c (HbA1c) at <7.5%, equivalent to 58 mmol/mol, which is recommended by the International Society for Pediatric and Adolescent Diabetes in children and adolescents (5). Achieving adequate metabolic control has proven to be a challenge worldwide. Therefore identifying possible predictors of metabolic control will be of benefit in adopting appropriate strategies employed to optimize outcomes.

Socioeconomic characteristics of patients are associated with glycemic control. Low socioeconomic class, single-parent family structure and lower parental supervision have been reported as predictors of poor metabolic control (6). Socioeconomic characteristics are variable among different communities and so it is crucial to assess these factors in diabetic children in each individual population, both in developed and developing countries, and to examine the association between those socioeconomic characteristics and metabolic control.

The aim of this study was to identify socioeconomic determinants of metabolic control in children with type 1 diabetes in a Jordanian population, which would help in developing appropriate strategies for diabetes care and education. In addition, it would facilitate the identification of high-risk patients who may require a more personalized management strategy.

## Methods

Data were collected by medical chart review of patients seen at pediatric endocrine clinics of two institutions: Jordan University Hospital and the National Centre for Diabetes, Endocrinology and Genetics, from February 2012 through December 2017. The University of Jordan Research Ethics Board approval was obtained (no: 51/2014-2015). Informed consent was not required as it is a chart review, retrospective study. Patients were eligible for the study if they had type 1 diabetes, age <18 years, and had at least one year of follow up.

Diagnosis of type 1 diabetes in our cohort was mainly based on the clinical picture. Variable antibodies were positive in variable percentages, with glutamic acid decarboxylase antibodies showing the highest propotion, followed by insulin autoantibodies and insulinoma antigen 2 antibodies. Antibody status supported the diagnosis of type 1 diabetes, but diagnosis was mainly based on clinical presentation with exclusion of patients with type 2 diabetes, maturity onset diabetes of the young and neonatal diabetes.

Body mass index (BMI) was used to categorize patients into two groups; normal and overweight, using the Centre for Disease Control and Prevention growth charts (7). Children with BMI values <85^th^ percentile were categorized as normal, and those with BMI values ≥85^th^ percentile were categorized as overweight.

Glycemic control was reflected by mean HbA1c values measured over the last year of follow up. Children with a HbA1c value of <7.5% (58 mmol/mol) were considered to be in metabolic control; while those with HbA1c ≥7.5% not in control. Dietary compliance was assessed by counting carbohydrates or determining portions.

When assessing the effect of place of residence, the participants were categorized into four groups depending on the distance from both centers, since they are adjacent to each other.

The socioeconomic status was expressed in terms of: 1) paternal and maternal level of education categorized into three groups - illiterate or elementary school, high school and bachelor’s degree or higher (Master’s degree and PhD); 2) paternal and maternal occupation was categorized into three groups - professional job, manual job and unemployed; and 3) total family monthly income was categorized into three groups: less than 400 Jordanian Dinars (JDs); which is the low income category, 400-800 JDs, and more than 800 JDs.

### Statistical Analysis

Statistical analysis was performed using IBM Statistical Package for the Social Sciences Statistics for Windows, version 23 (IBM Corp., Armonk, NY, USA).

Characteristics of the patients in the two metabolic groups, in control and not in control, were compared using Pearson chi-square and Fisher’s exact tests. Comparison of continuous variables among groups was conducted using One-Way ANOVA and Scheffe post-hoc analysis were used to identify the groups that were significantly different from each other.

Possible predictors of poor metabolic control were analyzed using multiple logistic regression (forward method). Metabolic control, expressed as HbA1c values categorized as a dichotomous nominal variable, was considered the dependent variable.

When logistic regression analysis was conducted, children with deceased mothers and/or fathers (11 cases) were excluded from regression analysis. We removed those cases to avoid large odds ratio (more than 6 digits) with inapplicable confidence intervals, a condition known as complete separation.

Multiple correspondence analysis (MCA) was conducted to analyze the association between socioeconomic characteristics and metabolic control. MCA allows researchers to analyze the pattern of relationships of several categorical dependent variables and detect underlying structures. P values less than 0.05 were considered statistically significant.

## Results

A total of two hundred and fifty-nine children with a mean ± standard deviation (SD) age of 11.14±3.61 years were enrolled in the study. One hundred and forty participants (54.1%) were males and 119 (45.9%) were females. Mean±SD HbA1c of the whole patient group was 8.77%±1.48% with 53 participants (20.5%) having an HbA1c level less than 7.5%, while HbA1c was ≥7.5% in two hundred and six children (79.5%).

The main characteristics of participants in the two metabolic groups are shown in Table 1. There were no significant differences between the two metabolic control groups concerning gender, age at diagnosis, type of insulin regimen and whether the child lived with both parents or with either one of them. Children who achieved their target HbA1c tended to have less than five siblings, normal BMI and adequate dietary compliance.

The socioeconomic characteristics are shown in Table 2. Among those, both maternal and paternal educational levels were significantly different between the two metabolic groups. Participants in the metabolically controlled group had a higher percentage of parents with a bachelor’s degree or higher. There were no significant differences between the two groups in terms of monthly income, parental job, parental age and parental medical condition.

The percentage of patients who experienced at least one episode of diabetic ketoacidosis, hospitalization and/or emergency room (ER) visits due to hyperglycemia in the metabolically-controlled group was significantly lower than in the metabolically-uncontrolled group: 0% *vs* 9.2% p=0.022; 3.8% *vs* 17.5%, p=0.012; and 0% *vs* 9.2%, p=0.022, respectively. However, hospitalization and ER visits due to hypoglycemia were not statistically different between the two groups: 0% *vs* 2.9%, p=0.209; and 1.9% *vs* 3.4%, p=0.571, respectively.

The association of the participants’ socioeconomic characteristics with metabolic control was investigated by conducting logistic regression analysis using the forward method (Table 3).

Poor metabolic control was associated with dietary non-compliance, being overweight, and low maternal educational level. Participants who were overweight were three and a half times more likely to have HbA1c ≥7.5% than those with normal weight. Patients with dietary non-compliance were almost four times more likely to experience poor metabolic control than those who were compliant. Participants whose mothers had a bachelor’s degree or higher were less likely to have poor metabolic control than those whose mothers were illiterate or had only elementary education [odds ratio (OR): 0.241, confidence interval (CI): 0.079 - 0.734; p=0.012].

**Figure 1:**
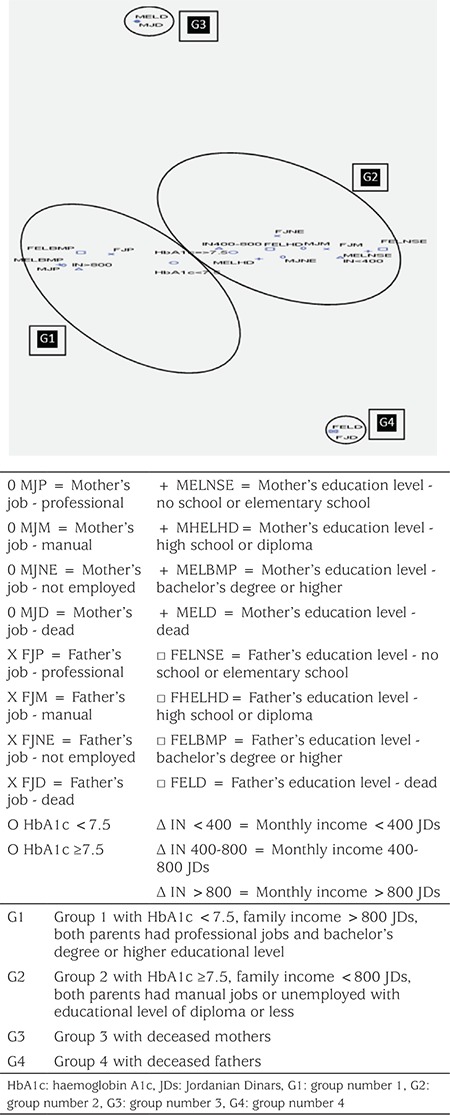
Association between socioeconomic factors and metabolic control in multiple correspondence analysis

MCA was conducted to explore patterns between socioeconomic status and metabolic control. The MCA model shown in Figure 1 explained 80% of total variability in the model and revealed four groups of patients. Group number 1 consisted of patients who were metabolically controlled, had a high family income, and both parents had professional jobs from the highest education level group. Group number 2 consisted of patients with poor metabolic control, low to intermediate monthly family income, and both parents had manual jobs or were unemployed with an education level lower than bachelor’s degree. Group number 3 (G3) consisted of patients whose mothers were deceased. Similarly, Group number 4 (G4) consisted of patients whose fathers were deceased.

Further analysis of the G3 showed that they had a significantly higher HbA1c of 10.6±1.86% compared to an average of 8.7±1.45% for the rest of participants (p=0.005). However, children whose fathers were dead also had a higher HbA1c that was statistically non-significant, 9.3±2.16% compared to 8.8±1.46% for the rest of the children (p=0.523).

## Discussion

The percentage of children who achieved target HbA1c (20.5%) was similar to that reported in studies reported from developed countries (8,9). Gender in our study was not associated with metabolic control, while evidence from studies concerning gender is conflicting (10,11,12). Longer duration of diabetes was found to be associated with poor metabolic control, similar to previous studies (13).

In our study, the MCA model revealed an association between low socioeconomic status and poor metabolic control, a finding which was also demonstrated in several previous studies (8,14,15,16). This effect even persists through adulthood where, in adults, low socioeconomic status was reported to increase mortality risk in patients with childhood-onset type 1 diabetes (17).

Poor metabolic control was associated with number of siblings exceeding four, which may be attributed to reduced attention and care that was provided to the patient by parents trying to manage a large number of children, a finding in agreement with previous reports (18).

The incidence of hypoglycemia was not significantly different in the controlled and the uncontrolled metabolic groups, while the rate of hyperglycemia was significantly higher in the metabolically uncontrolled group, again similar to previous reports (19). These results show that fear of hypoglycemia should not prevent families from achieving metabolic control for their children (20).

Monthly income was not significant in predicting metabolic control. This is in contrast to results from other studies that found a negative linear association between income and metabolic control (16,21,22). This association may be attributed to the fact that lower-income households rarely contact a primary care provider when facing a diabetes-related problem (23). However, in our study this lack of association between income and metabolic control can be explained by the fact that most of the subjects were covered by insurance and had access to the same level of diabetes care provided by specialized pediatric endocrinologists, regardless of family income. Distance between patient’s residence and the two adjacent diabetes centers, was not significantly related to metabolic control, probably due to the fact that 50% of our cohort lived in the same city and 86% of patients lived within 70 kilometers. In addition, both personal and public transport was relatively easily accessible to the families (11).

One of the socioeconomic characteristics predicting metabolic control in our study was parental educational level. The higher the maternal and paternal education the better the metabolic control. Paternal educational level in studies such as those from Italy and Saudi Arabia (8,24), was reported to have no effect on metabolic control. In our study the caregiver responsible for most of the diabetes care was the patient’s mother, even in the families where the patient lived with both parents (92% of our cohort). This is understandable since mothers are usually the primary caregivers coordinating efforts and medical recommendations provided by the members of the medical team. Improving mother’s knowledge and targeting those with lower educational level may improve glycemic control in this subgroup of children. Furhthermore, children whose mothers are dead; were found to have significant poor metabolic control since they are deprived of direct maternal care and supervision. It is extremely important to give those children special attention to optimize their diabetes care as much as possible with the help of their caregivers.

### Study Limitations

Our study was a retrospective study which mainly relied on information present in the medical records. One of the limitations of our study is that pubertal status was not collected from the medical charts. Another important limitation is the fact that the study involved patients registered in two centers in the capital city. A more comprehensive study involving different geographical areas with possible different socioeconomic determinants of metabolic control might have been more helpful in explaining the health inequalities that possibly exist in a society, despite the presence of the same policy of medical care. It is important to evaluate the effect of socioeconomic factors in both developed and developing countries to explain the universal challenge of achieving adequate metabolic control despite all the recent advancement in the field of diabetes care.

## Conclusion

Early metabolic control is essential in preventing future complications of type 1 diabetes. Identifying predictors of poor metabolic control might help in improving clinical care provided for patients.

Some predictors of poor glycemic control are modifiable, such as dietary non-compliance and being overweight, which can be controlled to improve glycemic control. Other predictors such as low maternal education level are non-modifiable, but these factors help in identifying those children and adolescents with type 1 diabetes at high risk of poor metabolic control. This group of children needs individualized care plans to ensure that target HbA1c levels are achieved. Children, whose mothers are dead, probably require special attention since they are at high risk of poor glycemic control. Engaging their caregivers and providing comprehensive education concerning the care plans of diabetic children is of great importance. These children might also need early, intensive and more frequent education and training on personal insulin requirements and administration since they lack maternal guidance and care.

## Figures and Tables

**Table 1:**
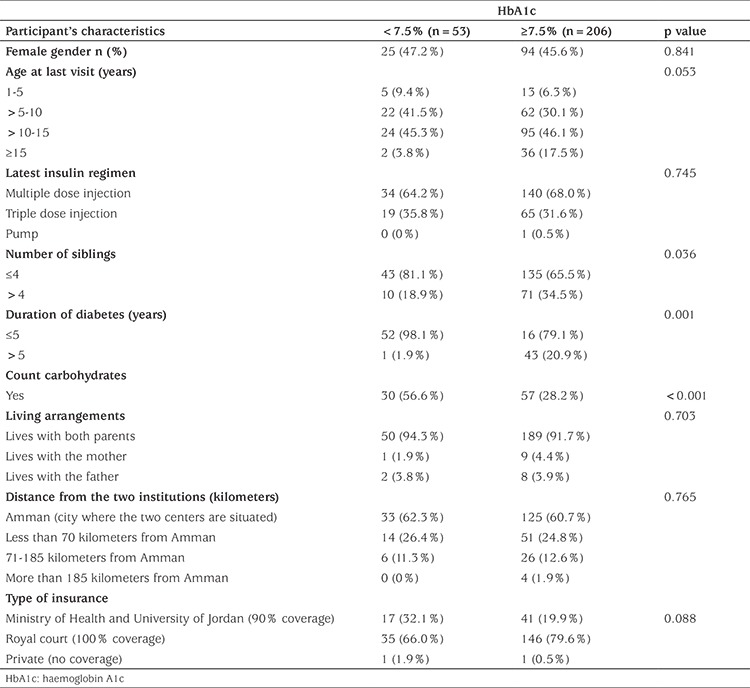
Characteristics of participants in the two metabolic control groups (n=259)

**Table 2:**
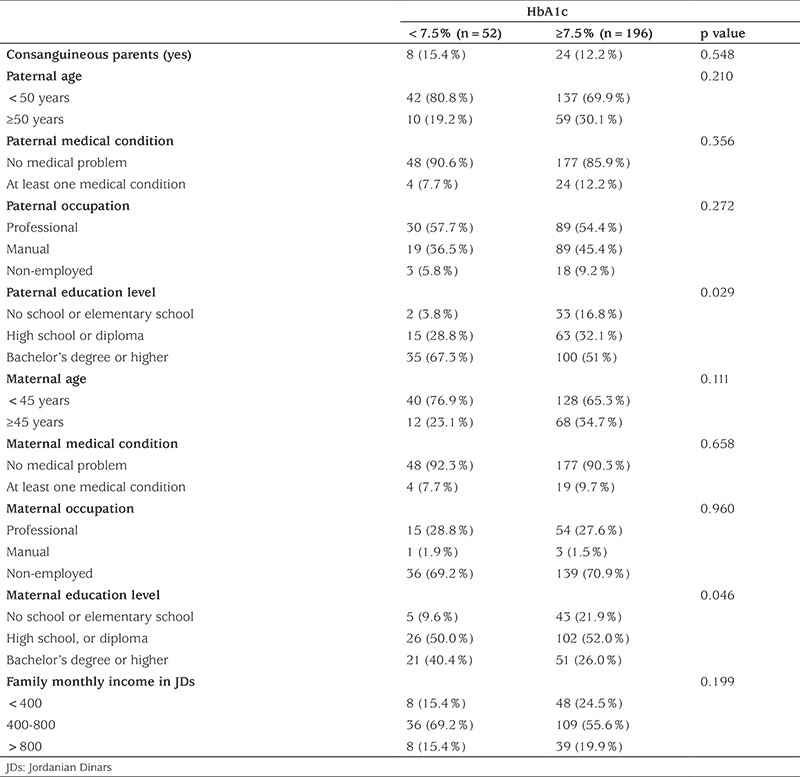
Parental characteristics of participants in the two metabolic control groups (n=248, 11 children with deceased father and/or mother were excluded)

**Table 3:**
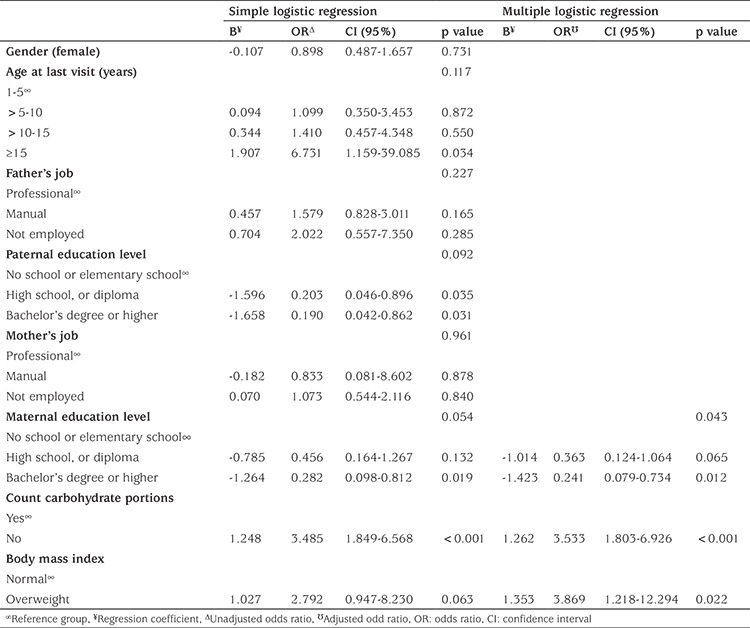
Logistic regression analysis of possible predictors of poor metabolic control (n=248; 11 children with deceased father and/or mother were excluded)
